# EpiDISH web server: Epigenetic Dissection of Intra-Sample-Heterogeneity with online GUI

**DOI:** 10.1093/bioinformatics/btz833

**Published:** 2019-11-09

**Authors:** Shijie C Zheng, Charles E Breeze, Stephan Beck, Danyue Dong, Tianyu Zhu, Liangxiao Ma, Wei Ye, Guoqing Zhang, Andrew E Teschendorff

**Affiliations:** 1 CAS Key Laboratory of Computational Biology, CAS-MPG Partner Institute for Computational Biology, Shanghai Institute of Nutrition and Health, Shanghai Institutes for Biological Sciences, University of Chinese Academy of Sciences, Chinese Academy of Sciences, Shanghai 200031, China; 2 Altius Institute for Biomedical Sciences, Seattle, WA 98121, USA; 3 UCL Cancer Institute, University College London, London WC1E 6BT, UK; 4 Bio-Med Big Data Center, CAS Key Laboratory of Computational Biology, CAS-MPG Partner Institute for Computational Biology, Shanghai Institute of Nutrition and Health, Shanghai Institutes for Biological Sciences, University of Chinese Academy of Sciences, Chinese Academy of Sciences, Shanghai 200031, China

## Abstract

**Summary:**

It is well recognized that cell-type heterogeneity hampers the interpretation of Epigenome-Wide Association Studies (EWAS). Many tools have emerged to address this issue, including several R/Bioconductor packages that infer cell-type composition. Here we present a web application for cell-type deconvolution, which offers the functionality of our EpiDISH Bioconductor/R package in a user-friendly GUI environment. Users can upload their data to infer cell-type composition and differentially methylated cytosines in individual cell-types for a range of different tissues.

**Availability and implementation:**

EpiDISH web server is implemented with Shiny in R, and is freely available at https://www.biosino.org/EpiDISH/.

## 1 Introduction

Epigenome-wide association studies (EWAS) aim to identify epigenetic alterations associated with disease, or with environmental risk factors. Most EWAS have been conducted in complex tissues (e.g. blood, buccal swabs) that are made up of many different cell-types. Since DNA methylation (DNAm) is highly cell-type specific, cell-type heterogeneity can lead to confounding (Jaffe and Irizarry, 2014; Liu *et al.*, 2013). To address this, many different cell-type deconvolution algorithms have been proposed (Houseman et al. 2012, 2014; Teschendorff and Relton, 2018). Previously, we developed a Bioconductor/R package named EpiDISH, which encompasses three ‘reference-based’ cell-type deconvolution algorithms and four DNAm reference matrices (Teschendorff *et al.*, 2017; Zheng *et al.*, 2018b). To enable usage of these methods for those unfamiliar with R programming, we here present a web server application, which provides a user-friendly interface and remote cloud computing capabilities. In addition to inferring cell-type fractions and offering interactive graphical output, we also include our CellDMC algorithm, which exploits the estimate cell-type fractions to identify differentially methylated cytosines (DMCs) in individual cell-types (DMCTs) (Zheng *et al.*, 2018a).

## 2 Description

**Fig. 1.**
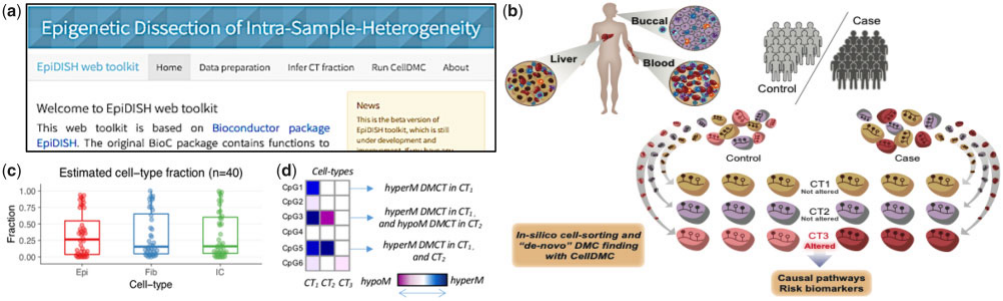
Graphical User Interface of the EpiDISH web server, conceptual idea of CellDMC and output examples. (**a**) The figure shows part of the home page of EpiDISH web server. As arranged in the navigator tabs, users can upload their data, infer cell-type fractions and run CellDMC to identify DMCTs in turn. (**b**) Conceptual idea of CellDMC. (**c**) Boxplots display estimated cell-type fractions of example dataset. (**d**) Examples of different types of DMCTs

The web server is depicted in Figure 1a, and consists of three main steps.

### 2.1 Data preparation

Users are required to upload their DNAm beta value matrix, phenotype of interest (POI) file (optional) and covariates matrix (optional). Both .txt and .csv formatted files will be accepted. After uploading, a preview will automatically appear on the right side of the page, so users can check that everything is ok. A prompt message will tell the users about the dimensions of the uploaded data.

### 2.2 Inference of cell-type fractions

We provide references for three tissues types, including whole blood, general epithelial tissues and breast tissue. For each of them the user can select the cell-types for which they would like to estimate fractions. For general epithelial and breast tissue, users can specify whether they would like to know the fractions of immune cell subtypes, as we otherwise return the total immune cell fraction. Users can also choose the estimation method from: robust partial correlation (Teschendorff *et al.*, 2017), constrained projection (Houseman *et al.*, 2012) or Cibersort (Newman *et al.*, 2015). Users can download the estimated cell-type fractions as .txt files. In addition, an interactive boxplot showing the estimated fractions will appear (Fig. 1c). Users can brush the boxplot to select several data points, with the names and fractions showing in a table below the boxplot. This feature allows users to quickly check outliers and spot potential problems. If the users upload a POI file, EpiDISH uses the reactive functionality of Shiny (RStudio Team, 2015) to generate another boxplot showing estimated fractions for each cell-type colored by POI. This boxplot can be downloaded as a .pdf file.

### 2.3 Running CellDMC

We can also identify the DMCs in each cell-type using CellDMC (Zheng *et al.*, 2018a) (Fig. 1b). The web server will first check whether the estimated cell-type fractions and POI are provided. If not, an error message will prompt. For CellDMC parameters, users can change the method to correct for multiple hypothesis testing, and the adjusted *P*-value threshold to call DMCTs (Fig. 1d). The DMCT results will be shown on the right side of the page in table format, which can be downloaded as .txt file. Users can also choose Illumina 450 K or EPIC array annotations to be included. In the interactive table you can search for a gene name, and the table will automatically be filtered for all CpGs mapped to that gene. This feature also applies to the estimated coefficients of each cell-type. The web tool also generates a downloadable scatter plot displaying the *t*-statistics between pairs of cell-types.

## 3 Conclusions

In summary, the EpiDISH server is a user-friendly toolkit for the EWAS community, offering cell-type fraction estimation and identification of differentially methylated cell-types.

## Funding

AET was supported by the Royal Society and Chinese Academy of Sciences (Newton Advanced Fellowship 164914) and the NSFC (grant numbers: 31571359, 31771464); L.M., W.Y. and G.Z. are supported by National Key R&D Program of China (grant numbers: 2017YFC0907505, 2016YFC0901904).

*Conflict of Interest*: none declared.
